# Provision of peri‐operative patient blood management strategies in the UK: a national survey of practice

**DOI:** 10.1111/anae.16579

**Published:** 2025-03-12

**Authors:** Samantha R. Warnakulasuriya, Kathleen Wolff, Simon J. Stanworth, of the Research and Audit Federation of Trainees group and the NIHR BTRU in Data Driven Transfusion Practice Collaborators, Hayley Evans, Alwyn Kotze, Mike Murphy, Antony Palmer, Akshay Shah, Noemie Roy, Martha Belete, William Spencer, Katie Preston, Rebecca Hawes, Reeanne Jones, Murray Williams, Ahmed Ibrahim, Aimi Lara Emilie Jeanes, Akshay Shah, Alan Race, Alexander Bell, Alison Chalmers, Alison Evans, Amarjeet Patil, Amelia Van Manen, Amy Thomas, Anandh Balu, Annette Haines, Annie Smith, Apurva Patil, Ashish Gandhi, Ashley McIlroy, Asya Veloso Costa, Barrie Philip Robertson, Beatrice Meilak, Ben Chandler, Bhavesh Gohil, Carlos Eduardo Abrunhosa de Mattos, Charlotte Katie Morris, Chintan Vora, Chiu Lee, Chris Oscier, Christopher Dixon, Ciara O'Brien, Clare Dallimore, Clare Quaterman, Colette Keenan, Cristian Lasai, Dale Seddon, Daniel Haslam, Darren Caldow, David Perry, Declan Love, Dina Bowey, Earlene Armstrong, Elizabeth Ribey, Emily Yeung, Emma Jacobs, Farzana Begum, Freya Brownlow, Gautam Dhananjay Modak, Gemma Talling, Georgia Monantera, Gomathy Kandasamy, Hannah Houston, Hannah Kennedy, Hannah Louise Headon, Helen Tyler, Henry Sergeant, Ismaa Aslam, Jade Loughran, Jake Melhuish, James Kirkland, James Morris, Jane Doherty, Jennifer Brooke, Jennifer Phillips, Jessica Irwin, Joel Prescott, Jordan McVey, Josephine Barnsley, Ka Po Tam, Katherine Saunders, Kim Rhodes, Krupa Basavaraj, Kush Amin, Laura Carter, Laura Field, Laura Fulton, Laura Scott, Leon Cohen, Louis Murphy, Madeleine Edwards, Madhavi Gudipati, Magde Albarade, Mai Wakatsuki, Manaf Al‐Bayati, Mark Dorrance, Marta Malaj, Matt Bridge, Matthew Peacock, Maya Sussman, Michael Jarvis, Michael Olivier, Mohamed Elbahnasy, Mohammad Sharif, Molly Janowski, Nadeeshya Dulanjalee Welikala, Naomi R C Adey, Nicola Coverdale, Noorunisa Suhail, Oliver Dare, Orlagh McNally, Paul Young, Pei Shan Lim, Peter Sandbach, Philippa Horne, Prathiban Kumar, Puja Chhaniyara, Putri Rimba, Rabia Ghani, Rashmi Rebello, Rebecca Allott, Rebecca Vickers, Richard Timoney, Rishi Naik, Robert Davidson, Robert Palin, Rosanna Seatter, Rotimi Latinwo, Ruchi Maniar, Ryad Sammy Chebbout, Sabba Aziz, Samah Alimam, Samuel Lichfield, Sharon Ramirez, Shashiharen Gnanapandithen, Shashikant Yegnaram, Shriyam Patel, Simon Beecroft, Sioned Elin Davies, Stephanie Worrall, Stuart Cleland, Subhadra Devi Balakrishnan, Suji Pararajasingam, Sumithre Gunathilake, Svetlana Kulikouskaya, Tajwinder Singh Sandhar, Tina Vaz, Toby Reynolds, Tom Kingston, Val Luoma, William Booth, Xiaosu Jiang, Zaid Saghir Ahmed, Zwesty Viera

**Affiliations:** ^1^ Department of Anaesthesia and Perioperative Medicine University College London Hospital NHS Foundation Trust London UK; ^2^ NIHR Blood and Transplant Research Unit in Data Driven Transfusion Practice (BTRU) University of Oxford UK; ^3^ Research and Audit Federation of Trainees (RAFT) UK; ^4^ Department of Anaesthetics University Hospitals of Leicester NHS Trust Leicester UK; ^5^ Department of Clinical Haematology Oxford University Hospitals NHS Foundation Trust Oxford UK; ^6^ NHS Blood and Transplant Oxford UK

**Keywords:** blood transfusion, iron, patient blood management, peri‐operative, tranexamic acid

## Abstract

**Introduction:**

In UK hospitals, it is unclear how organisational structures are arranged to support effective implementation of peri‐operative blood management practice strategies. The aim of this study was to conduct a national survey of organisations to describe local practices of peri‐operative patient blood management and infrastructure availability in the UK.

**Methods:**

A series of benchmarking standards was developed using recommendations informed by national standards, relevant literature and an expert panel. Through the Research and Audit Federation of Trainees networks, 143 hospitals were approached to participate. The pre‐piloted survey was conducted online between January and February 2023.

**Results:**

Responses were received from 123 hospitals across 74 NHS Trusts and health boards. Formal elective anaemia pathways were not reported in 37/123 (30%) sites. There was considerable inter‐site variation in interventional thresholds for anaemia and screening tests. A variety of oral iron regimens were reported, from once‐daily dosing in 41/85 (48%) sites, to three times a day dosing in 14/85 (16%). Ferric carboxymaltose was the preparation used most frequently at sites that administered intravenous iron (61/113, 54%). There was variation between hospitals and surgical specialties in the use of tranexamic acid with 49/122 (39%) hospitals reporting a policy for the use of peri‐operative tranexamic acid. For sites that performed major surgery routinely (irrespective of specialty), 20/112 (18%) included tranexamic acid in operating theatre safety briefings. Point‐of‐care coagulation testing was available at 62/123 (50%) sites.

**Discussion:**

Our findings show considerable heterogeneity in peri‐operative patient blood management strategies and supporting infrastructure availability across the UK. There is a pressing need for hospitals to review pathways of care offered to surgical patients and implement national recommendations.

## Introduction

Peri‐operative patient blood management requires a multidisciplinary, evidence‐based approach to optimise the care of patients who may require peri‐operative blood transfusion [1, 2]. There are existing recommendations [1, 3, 4] to reduce inappropriate use of blood components; minimise transfusion‐related complications; and ensure security of blood supply to patients when there is no alternative to transfusion [1, 5]. Despite evidence of benefit and safety [6, 7, 8] and the presence of national guidance in the UK [3, 9, 10], the uptake of peri‐operative blood management strategies within routine clinical practice remains unclear.

Relevant blood management strategies should be considered for each stage of the peri‐operative journey. Pre‐operative optimisation of anaemia, present in 30% of patients who require major elective surgery [11, 12], is a core blood management intervention [3]. Pre‐operative anaemia is associated with increased duration of stay [11], and morbidity [12], but transfusion may worsen outcomes [13, 14]. Intra‐operative patient blood management strategies include adoption of restrictive red blood cell transfusion thresholds; administration of tranexamic acid; use of cell salvage; and point‐of‐care haemoglobin and coagulation testing [3]. International uptake of patient blood management strategies has been described [6, 7, 15, 16], but in UK national audit data [17] and observational research [18], persistent gaps have been observed. These include inadequate pre‐operative optimisation despite severe pre‐operative anaemia [18] and the lack of tranexamic acid administration in approximately one‐third of patients having major surgery [17].

Clinicians and policy makers need to benchmark how peri‐operative blood management strategies are implemented at the hospital level across the UK to monitor and improve practice. These issues have taken added importance in the context of the UK infected blood inquiry report [19] and recent declared blood shortages [20]. The primary aim of this national survey was to describe the practices of peri‐operative patient blood management practice at hospitals across the UK. The focus was pre‐operative anaemia, but additional strategies of interest included intra‐operative interventions for minimising blood loss; adoption of appropriate defined transfusion thresholds; administration of tranexamic acid; use of cell salvage; and point‐of‐care haemoglobin and coagulation testing [3].

## Methods

Ethical approval was not required to conduct this study as determined by the NHS Health Research Authority Tool (see online Supporting Information Appendix S2). Additional local approvals at participating sites were gained as required. No patient level data were collected as a part of this study. This survey was conducted as a collaboration between the Research and Audit Federation of Trainees (RAFT) and the NIHR Blood and Transfusion Research Unit in Data Driven Transfusion Practice (BTRU) using REDCap (Research Electronic Data Capture). The RAFT is a national trainee research network, supporting UK‐wide delivery of research and quality improvement by postgraduate anaesthetists in training. The BTRU is a group of clinical and academic researchers (inclusive of patient representation) who use data‐driven methods to optimise use of blood and alternatives to transfusion to improve patient outcomes.

Survey design was informed through review of national standards [2, 3, 9, 10, 21, 22, 23, 24, 25] and relevant literature [26, 27, 28] by an expert panel. A series of benchmarking standards were developed from commonly accepted recommendations and guidance (summarised in Table 1 with further detail provided in online Supporting Information Table S1). The survey was piloted among six members of the data‐driven BTRU engagement panel (including NHS consultants, clinical academics with a background in anaesthesia and peri‐operative medicine, surgery and haematology) and seven members of the RAFT committee (resident doctors in anaesthesia), as representative of target respondents. Feedback from this piloting exercise was used to refine the survey questions which were then subject to further peer review by the BTRU before dissemination nationally. The finalised survey questions are available in online Supporting Information Appendix S3. The survey tool was designed with embedded logic to stream respondents to specific questions depending on the presence or absence of key local peri‐operative blood management services and guidance.

**Table 1:** Benchmark standards used for survey development.

Guidance	Pre‐operative	Intra‐operative
WHO Patient Blood Management Pillar [2]	Detection and management of anaemia and iron deficiency	Minimisation of blood loss and optimisation of coagulation
NICE Quality Standard QS138 [9] NICE Guidance NG24 [3]	Offer iron supplementation for patients with iron deficiency anaemia	Offer TXA if moderate blood loss expected
NICE Guidance NG45 [21]	Full blood count pre‐operatively for intermediate surgery and ASA 3–4 and all major surgery	
CPOC Standards of Care [22]	Full blood count at referral to surgery or at first surgical consultation if fulfils NICE NG45 criteria [21]	
CPOC Recommendations [10]	Haemoglobin thresholds to diagnose anaemia Assess causes of newly identified anaemia Hospital guideline Clinical lead for patients with peri‐operative anaemia	TXA 1 g intravenous before skin incision if expected blood loss > 500 ml (unless recent stroke or myocardial infarction)
Association of Anaesthetists [23]		Cell salvage use if blood transfusion or severe postoperative anaemia anticipated Cell salvage equipment and trained staff immediately available 24 h a day
GIRFT [24]	Early access to haemoglobin levels haematinics Effective pathways	Cell salvage systems are available when required in all surgical specialities
Joint Royal Colleges Tranexamic Acid in Surgery Implementation Group [25]		TXA considered for in‐patient surgery, 1 g of intravenous TXA at the start and end of surgery
BTRU expert consensus		Oral iron dosing Availability of point‐of‐care testing and rapid infusion devices

WHO, World Health Organization; NICE, National Institute for Health and Care Excellence; TXA, tranexamic acid; CPOC, Centre for Perioperative Care; GIRFT, Getting it Right First Time; BTRU, Blood and Transplant Research Unit in Data Driven Transfusion Practice.

The survey was circulated via the RAFT network with invitations to take part shared among RAFT regional trainee research networks and by contacting College Tutors and Heads of School of Anaesthesia in the region without RAFT representation. Collaborators were invited to complete the survey on behalf of hospital sites (hospital sites within the same NHS Trust were defined as separate sites if they were located over a mile apart from each other). Collaborators were provided with an invitation to complete a password‐protected entry hosted within REDCap. Reminders were sent weekly until either the survey was opened or when the survey window ended. No financial incentives were offered for completion of the survey and site lead collaborators are acknowledged in online Supporting Information Appendix S1.

We undertook a descriptive analysis of completed survey responses. Missing or incomplete data were cross‐checked with sites. Surveys that remained incomplete after this process were excluded to minimise missing data. Analysis was undertaken in Microsoft Excel (Microsoft Corporation, Redmond, WA, USA) and R (RStudio 2021.09.0+351 and 2023.09.0+463; R Project for Statistic Computing, Vienna, Austria), and summary statistical code was used to conduct simple descriptive analysis of data. This article has been prepared to comply with CROSS checklist for survey reporting [29] (online Supporting Information Table S2).

## Results

**Figure 1:**
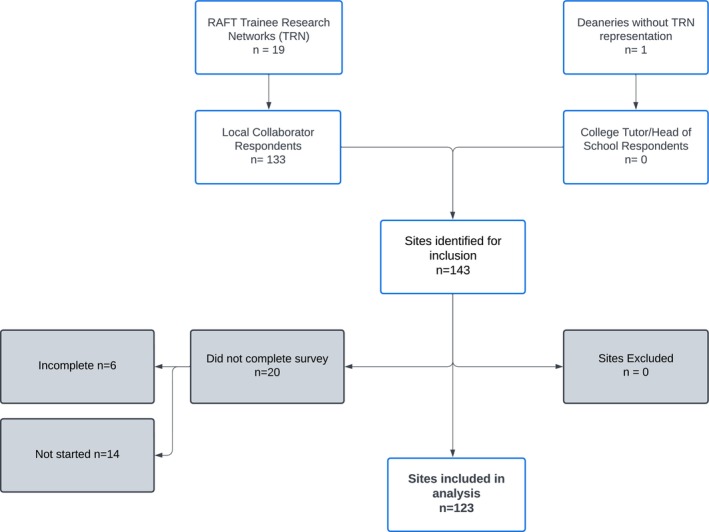
Site identification and recruitment to survey.

**Figure 2:**
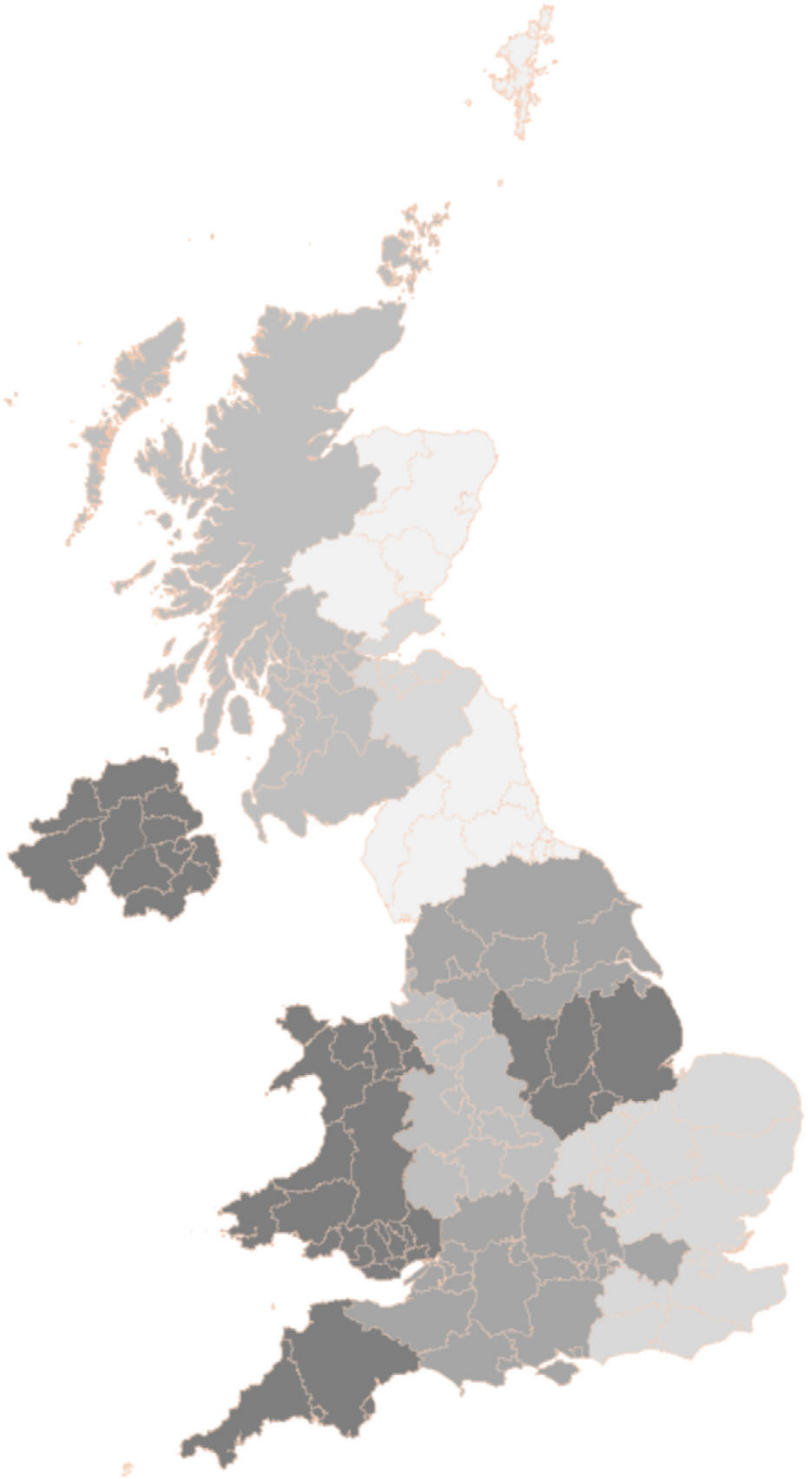
A geographical representation of the proportional response rates of sites per Trainee Research Network (School of Anaesthesia) [30]. Darker shades indicate higher response rates. Highest response rates (76–100%) MERCAT (East Midlands); NITECAP (Northern Ireland); SWARM (Peninsula); and WAAREN (Wales). Lowest response rate 0% CRANE (Northern); SEQUOIA (East of Scotland); No TRN (North Scotland).

The survey was conducted between 1 January and 28 February 2023. There were responses from 123 hospital sites spanning 74 NHS Trusts and health boards from 143 hospitals approached, with an overall completion rate of 86% (Fig. 1). The geographical spread of responding sites is shown in Fig. 2 and hospital characteristics are shown in Table 2. Most surveyed hospitals were inpatient centres (111/123, 90%) with a median (IQR [range]) bed number of 565 (400–789 [38–1594]), inclusive of a broad range of surgical specialities (Table 3 and online Supporting Information Table S3).

**Table 2:** Responding hospital site characteristics (n = 123). Values are number (proportion).

**Number of inpatient beds**	
Day case units	12 (10%)
≤ 500	51 (41%)
501–999	50 (41%)
≥ 1000	10 (8%)
**Hospital departments and infrastructure**	
Presence of emergency department	86 (70%)
Presence of ICU	97 (79%)
Presence of emergency theatres	94 (77%)
Presence of blood bank	104 (85%)
Ability to remote issue blood	45 (37%)

**Table 3:** Specialty level variation in routine tranexamic acid use and use of checklists by hospital site. Values are number (proportion).

Surgical specialty	Routinely use prophylactic tranexamic acid when indicated	Tranexamic acid on surgical safety checklists when indicated
Elective orthopaedics (n = 83)	66 (80%)	36 (43%)
Major trauma (n = 18)	14 (78%)	8 (44%)
Orthopaedic trauma (n = 80)	60 (75%)	40 (50%)
Cardiac (n = 22)	15 (68%)	9 (40%)
Neurosurgery (n = 15)	9 (60%)	5 (33%)
Transplant surgery (n = 19)	11 (58%)	7 (37%)
Major gynaecology cancer (n = 63)	35 (56%)	12 (19%)
Obstetrics (n = 76)	42 (55%)	27 (36%)
Thoracic (n = 22)	12 (55%)	6 (27%)
Burns and plastics (n = 23)	11 (48%)	5 (22%)
Major urology cancer (n = 56)	25 (45%)	12 (21%)
Upper gastrointestinal (n = 68)	26 (38%)	12 (18%)
Hepatobiliary (n = 32)	12 (38%)	10 (31%)
Major head and neck cancer (n = 38)	14 (37%)	10 (26%)
Lower gastrointestinal (n = 92)	33 (36%)	18 (20%)
Vascular (n = 33)	12 (36%)	9 (27%)

The main findings for each domain of blood management and are benchmarked against our a priori definitions are shown in Table 1 (full details in online Supporting Information Table S1). Overall, 86 (70%) sites reported implementation of a formal elective anaemia pathway as recommended by the Centre for Perioperative Care [10]; 66 (54%) reported a universal pathway to cover all surgical specialities offered locally; and 20 (16%) reported surgical specialty specific pathways. Anaesthetists were the most common professional group who manage formal peri‐operative anaemia pathways, in 77/86 (90%) of sites with this service.

Screening for anaemia was reported to occur at several time points during the patient pre‐operative pathway (Table 1 and online Supporting Information Table S1). The most common assessments were reported in a pre‐operative assessment clinic > 6 weeks before surgery in 87 (71%) sites, followed by pre‐operative assessment clinics < 6 weeks before surgery in 83 (67%) sites. There was overlap with 57 sites that selected both these options. The overall responses suggest that 68 (55%) of sites did not meet the National Institute for Health and Care Excellence guidance on timing of full blood count [21] (online Supporting Information Table S4) and reveals missed early opportunities for checking haemoglobin, such as point of referral for surgery; first surgical consultation; or when listing for surgery [10, 21].

Only 19 (15%) sites performed all Centre for Perioperative Care suggested blood tests routinely [10] to further identify causes of pre‐operative anaemia in patients diagnosed (Table 4). While the majority of sites reported checking ferritin (110, 89%), transferrin saturation (97, 79%), B12 and folate (105, 85%), reticulocyte haemoglobin content was only checked at 24 (20%) sites. When sites estimated the proportion of anaemic patients who underwent these supplementary investigations, 59 (48%) sites indicated < 80% of patients received further investigation with 32 (26%) of sites reporting it was undertaken in < 50% of patients. Referral thresholds for anaemia by haemoglobin varied between hospital sites. Over two‐thirds of sites (86, 70%) described referral pathways with specific thresholds; and in these cases, 41 (48%) reported a threshold of < 130 g.l^‐1^ for females and males; 31 (36%) a threshold of < 130 g.l^‐1^ for males and < 120 g.l^‐1^ for females; and 14 (16%) used other referral thresholds.

**Table 4:** Reported use by site for recommended pre‐operative blood tests [10] (n = 114; nine sites excluded where data were missing). Values are number (proportion).

Test	Yes	No	Unsure
Reticulocyte haemoglobin content	24 (21%)	69 (61%)	21 (18%)
Reticulocyte count	40 (35%)	58 (51%)	16 (14%)
B12/folate	105 (92%)	8 (7%)	1 (< 1%)
Renal function	89 (78%)	15 (13%)	10 (9%)
C‐reactive protein	74 (65%)	30 (26%)	10 (9%)
Transferrin saturation	97 (85%)	14 (12%)	3 (3%)
Serum ferritin	110 (96%)	3 (3%)	1 (1%)

In total, 85 (69%) hospitals reported local guidance for oral iron supplementation. However, a variety of iron regimens were reported, including once daily dosing (41, 48%); alternate day dosing (28, 33%); twice per day dosing (24, 28%); and three times per day dosing (14 (16%)). Intravenous iron administration was reported by 113 (92%) sites; ferric carboxymaltose was the most frequent formulation used (61, 54%), followed by ferric derisomaltose (47, 41%). Most sites reported that iron infusions were typically administered in medical ambulatory units or outpatient settings. Further information on anaemia screening and treatment is shown in the online Supporting Information Table S4.

Intra‐operative use of tranexamic acid was evaluated in comparison with benchmarking standards (Table 1 and online Supporting Information Table S1). Table 3 shows a summary of responses regarding availability of local policies for the use of intra‐operative tranexamic acid. Eleven sites reported no indication for use of tranexamic acid as they did not perform surgery associated with moderate to high blood loss leaving 112 reporting sites [9]. Less than half of hospital sites (49, 44%) reported a policy for the use of peri‐operative intravenous tranexamic acid. Of the 49 hospitals that had a tranexamic acid policy, 21 (43%) reported that their policies indicated an exclusion criterion such as stroke or recent myocardial infarction (online Supporting Information Table S5). Of the 112 of sites that performed surgery associated with moderate to high blood loss, only 20 (18%) included tranexamic acid during operating theatre safety briefings. Prophylactic tranexamic acid was not routinely offered at 19 (17%) sites but, despite gaps in formal policy, it was used routinely across all surgical specialties at 36 (32%) of sites and in specific surgical specialties at 57 (51%) sites. The most commonly reported dose of tranexamic acid used was 1 g intravenously (see online Supporting Information Table S5). Differences in rates of routine tranexamic acid use depending on surgical specialty are shown in Table 3.

Point‐of‐care coagulation testing was available at 50% (62/123) of sites. Thirty‐seven per cent (45/123) of sites reported availability of remote blood issuance (a process for obtaining timely blood components from a blood fridge if operating theatres are positioned remotely from the blood bank). There was variation in the availability of rapid infusers and point‐of‐care coagulation tests dependent on specialty and hospital size (Table 5). Local policies for the use of cell salvage were not present at 59% (73/123) of sites overall, and not present at 34% (26/76) of sites offering obstetric services. At sites with local policies, the most common organisational level indication for cell salvage was patient refusal of allogeneic transfusion for both obstetric and non‐obstetric patients (see online Supporting Information Table S6).

**Table 5:** Availability of patient blood management infrastructure for inpatient hospitals stratified by number of beds (excluded day case units (n = 12) and no bed data (n = 2)). Values are median (IQR [range]) and number (proportion).

Hospital size	Number of sites	Number of beds	Sites with available infrastructure			
			Cell salvage machine	Rapid infuser	Point‐of‐care coagulation testing	Remote issue ability
> 500 beds	59	770 (640–900 [521–1594])	55 (93%)	55 (93%)	39 (66%)	20 (34%)
≤ 500 beds (excluding day case)	50	361 (203–457 [38–500])	36 (72%)	40 (80%)	21 (42%)	22 (44%)
Total	109	565 (400–789 [38–1594])	91 (83%)	95 (87%)	60 (55%)	42 (39%)

## Discussion

We found a lack of formal pathways to guide management of pre‐operative anaemia at 30% of hospital sites; variability in the timing of assessments for anaemia; inconsistency in haemoglobin thresholds for anaemia optimisation; limited policies for the investigation and management of anaemia, including use of oral iron; underuse of tranexamic acid; and a lack of policies for use of cell salvage. This is important because implementation of blood management recommendations is known to improve patient outcomes and reduce transfusion rates [6]. We recommend that regular national audits are conducted to benchmark hospital practice and drive quality improvement for patients at risk of peri‐operative bleeding and blood transfusion.

Blood management optimisation requires clarity on definitions of anaemia. Our findings are consistent with existing literature that reports varied and inconsistent guidance between organisations [10, 26, 31]; diagnostics [10, 26, 32]; and dose strategies for oral iron [28, 33]. We observed that oral iron administration policies were not in line with recent evidence to optimise dosage and uptake [28], reflecting a translation gap for a key component of anaemia management. Access to intravenous iron was reported widely in our survey, although the evidence base for use (including cost‐effectiveness) remains unclear [11, 12, 34, 35, 36, 37]. Overall, our findings highlight that peri‐operative anaemia management strategies should be prioritised to meet with NHS England priorities for elective care [38]. This requires patients to be monitored in effective peri‐operative pathways and screened for peri‐operative risk factors as early as possible in their surgical pathway [38].

The importance of blood transfusion alternatives was highlighted recently in the UK‐infected blood inquiry report [39]. Our findings suggest that tranexamic acid is likely to be underutilised in patients who undergo surgery with anticipated moderate to high blood loss, with evidence of variation in use according to surgical specialty. Targeted quality improvement efforts must be a priority for sites, to promote increased uptake of tranexamic acid at a hospital level [25, 40]. There was considerable variation between sites in access to infrastructure for other blood management interventions, such as cell salvage or point‐of‐care testing. Point‐of‐care testing may allow targeted intra‐operative transfusion. All sites undertaking cardiac surgery reported access to point‐of‐care coagulation testing, but only 50% of sites that perform obstetric surgery reported access. A significant proportion of sites that had access to cell salvage machines did not have specific policies for use. Limited cell salvage usage due to concerns regarding infection, cancer and haemoglobinopathies, considered as relative contraindications by the UK Cell Salvage Action Group [41], require further evaluation [42].

Our survey was designed using a multidisciplinary approach with input from anaesthetists, surgeons and haematologists, drawing on guidance for surveys [29]. Sites from the organisational survey were defined using a similar methodology to SuperSNAP1 [43] with responding sites representative of UK hospital sizes (Table 5) and geography (Fig. 2). Our results, therefore, encompass practice across the full range of NHS services where major inpatient surgery is undertaken. The use of a survey format allows exploration of ‘real world’ implementation that can be compared with the research base and national policy and guidance. Limitations include those common to all surveys, including the failure to reflect actual practice [44] or the practice of non‐respondents. Our survey is reliant on collaborator knowledge of pathways. RAFT collaborators, who are likely to be rotational employees, may not be aware of all policies and practices. To address this challenge, RAFT collaborators were encouraged to seek information from substantive staff and have a named senior supervisor. Surveys that remained incomplete after cross‐checking were excluded to minimise missing data. This process established that a single question that asked for the approximate number of elective surgical operations per year was difficult for local collaborators to answer accurately and so we have excluded these answers from analysis. However, despite these limitations, overall, our findings appear consistent with published data including the National Comparative Audit of Blood Transfusion [17] and Getting it Right First Time [24].

In summary, our survey highlights multiple areas where local provision of peri‐operative services should be improved, to enable focused implementation of established national guidance on patient blood management aimed at improving the care of patients requiring surgery. Our survey design has established a baseline which can be used to generate targets for future quality improvement or research interventions. This baseline will facilitate practice benchmarking, highlighting where there is a lack of provision of local policies or pathways, and to identify areas for healthcare spending. Unanswered research questions include topics such as identifying which patients benefit from intravenous iron therapy and cell salvage; research in these areas would allow implementation of cost‐effective, patient‐centred optimisation of patient blood management in the future. We recommend that this survey is repeated on an annual basis to benchmark implementation of peri‐operative blood management strategies and drive improved care for patients.

## Supporting information


**Appendix S1.** Collaborators list.

**Appendix S2.** HRA decision tool outcome.

**Appendix S3.** Survey questions.

**Table S1.** Benchmarking standards with mapped results.
**Table S2.** Checklist for Reporting Of Survey Studies (CROSS).
**Table S3.** Surgical specialties.
**Table S4.** Organisational survey questions and responses for questions relating to peri‐operative anaemia pathways for elective surgery.
**Table S5.** Organisational survey questions relating to tranexamic acid use.
**Table S6.** Organisational questions relating to cell salvage.
