# Supplementary material for: Provision of peri‐operative patient blood management strategies in the UK: a national survey of practice
Source: Anaesthesia. 2025 Mar 12;80(7):781–9. doi: 10.1111/anae.16579 (PMC12171794; doi:10.1111/anae.16579)
Supplement: Supplementary file 1 — Appendix S1. Collaborators list. [file ANAE-80-781-s003.docx]

**Appendix S1** Key collaborators

Hayley Evans

Alwyn Kotze

Mike Murphy

Antony Palmer

Akshay Shah

Noemie Roy

Martha Belete

William Spencer

Katie Preston

Rebecca Hawes

Reeanne Jones

Murray Williams

Ahmed Ibrahim

Aimi Lara Emilie Jeanes

Akshay Shah

Alan Race

Alexander Bell

Alison Chalmers

Alison Evans

Amarjeet Patil

Amelia Van Manen

Amy Thomas

Anandh Balu

Annette Haines

Annie Smith

Apurva Patil

Ashish Gandhi

Ashley McIlroy

Asya Veloso Costa

Barrie Philip Robertson

Beatrice Meilak

Ben Chandler

Bhavesh Gohil

Carlos Eduardo Abrunhosa de Mattos

Charlotte Katie Morris

Chintan Vora

Chiu Lee

Chris Oscier

Christopher Dixon

Ciara O'Brien

Clare Dallimore

Clare Quaterman

Colette Keenan

Cristian Lasai

Dale Seddon

Daniel Haslam

Darren Caldow

David Perry

Declan Love

Dina Bowey

Earlene Armstrong

Elizabeth Ribey

Emily Yeung

Emma Jacobs

Farzana Begum

Freya Brownlow

Gautam Dhananjay Modak

Gemma Talling

Georgia Monantera

Gomathy Kandasamy

Hannah Houston

Hannah Kennedy

Hannah Louise Headon

Helen Tyler

Henry Sergeant

Ismaa Aslam

Jade Loughran

Jake Melhuish

James Kirkland

Jane Doherty

Jennifer Brooke

Jennifer Phillips

Jessica Irwin

Joel Prescott

Jordan McVey

Josephine Barnsley

Ka Po Tam

Katherine Saunders

Kathleen Wolff

Katie Preston

Kim Rhodes

Krupa Basavaraj

Kush Amin

Laura Carter

Laura Field

Laura Fulton

Laura Scott

Leon Cohen

Louis Murphy

Madeleine Edwards

Madhavi Gudipati

Magde Albarade

Mai Wakatsuki

Manaf Al-Bayati

Mark Dorrance

Marta Malaj

Matt Bridge

Matthew Peacock

Maya Sussman

Michael Jarvis

Michael Olivier

Mohamed Elbahnasy

Mohammad Sharif

Molly Janowski

Nadeeshya Dulanjalee Welikala

Naomi R C Adey

Nicola Coverdale

Noorunisa Suhail

Oliver Dare

Orlagh McNally

Paul Young

Pei Shan Lim

Peter Sandbach

Philippa Horne

Prathiban Kumar

Puja Chhaniyara James Morris

Putri Rimba

Rabia Ghani

Rashmi Rebello

Rebecca Allott

Rebecca Vickers

Reeanne Jones

Richard Timoney

Rishi Naik

Robert Davidson

Robert Palin

Rosanna Seatter

Rotimi Latinwo

Ruchi Maniar

Ryad Sammy Chebbout

Sabba Aziz

Samah Alimam

Samantha Warnakulasuriya

Samuel Lichfield

Sharon Ramirez

Shashiharen Gnanapandithen

Shashikant Yegnaram

Shriyam Patel

Simon Beecroft

Sioned Elin Davies

Stephanie Worrall

Stuart Cleland

Subhadra Devi Balakrishnan

Suji Pararajasingam

Sumithre Gunathilake

Svetlana Kulikouskaya

Tajwinder Singh Sandhar

Tina Vaz

Toby Reynolds

Tom Kingston

Val Luoma

William Booth

Xiaosu Jiang

Zaid Saghir Ahmed

Zwesty Viera
