# Supplementary material for: Provision of peri‐operative patient blood management strategies in the UK: a national survey of practice
Source: Anaesthesia. 2025 Mar 12;80(7):781–9. doi: 10.1111/anae.16579 (PMC12171794; doi:10.1111/anae.16579)
Supplement: Supplementary file 3 — Appendix S3. Survey questions. [file ANAE-80-781-s002.pdf]

Perioperative Blood Management: Organisational Survey

This survey is designed to be completed for sites that provide adult surgical services which require anaesthetic input. Sites that exclusively do paediatrics are not included in this project.

The first survey you complete should cover the main site within your trust unless you have a co-lead completing this already. A separate survey should then also be completed for any other sites within the trust that provide surgical services with anaesthetic support that are >1 mile away from the main site. Hospital details will only be used to determine if there are any differences between sites and hospitals or trusts will be not be identifiable in any future publications.

This survey link is unique to your hospital site and should not be shared between sites. If you are site lead for more than one site you should have recieved more than one survey link, if not please contact us and we will send you the number unique survey links you require.

You can save the survey and return to it later to complete it at a later point. Once completed and submitted you will not be able to make any changes to the form without contacting us first. The deadline for survey submission is 31st January 2023.

Once you have submitted the organisational survey we will send out certificates within 1 month.

You can find a blank pdf copy of this survey on our website:
https://www.raftrainees.org/perioperative-blood-management

For any questions or further advice please contact us on: pbm.raft@gmail.com

Thank you for your participation!

Site Information

Name of NHS Hospital Trust / local health board

Start typing the name of your trust or health board and it should come up, if not select other and free text underneath

- ◯ Airedale NHS Foundation Trust
- ◯ Ashford and St Peter's Hospitals NHS Foundation Trust
- ◯ Barking, Havering and Redbridge University Hospitals NHS Trust
- ◯ Barnet, Enfield and Haringey Mental Health NHS Trust
- ◯ Barnsley Hospital NHS Foundation Trust
- ◯ Barts Health NHS Trust
- ◯ Bedford Hospital NHS Trust
- ◯ Bedfordshire Hospitals NHS FoundationTrust
- ◯ Berkshire Healthcare NHS Foundation Trust
- ◯ Birmingham and Solihull Mental Health NHS Foundation Trust
- ◯ Birmingham Community Healthcare NHS Foundation Trust
- ◯ Birmingham Women's and Children's NHS Foundation Trust
- ◯ Black Country Healthcare NHS Foundation Trust
- ◯ Blackpool Teaching Hospitals NHS Foundation Trust
- ◯ Bolton NHS Foundation Trust
- ◯ Bradford District NHS Foundation Trust
- ◯ Bradford Teaching Hospitals NHS Foundation Trust
- ◯ Bridgewater Community Healthcare NHS Foundation Trust
- ◯ Buckinghamshire Healthcare NHS Trust
- ◯ Burton Hospitals NHS Foundation Trust
- ◯ Calderdale and Huddersfield NHS Foundation Trust
- ◯ Cambridge University Hospitals NHS Foundation Trust
- ◯ Cambridgeshire and Peterborough NHS Foundation Trust
- ◯ Cambridgeshire Community Services NHS Trust
- ◯ Camden and Islington NHS Foundation Trust
- ◯ Central and North West London NHS Foundation Trust
- ◯ Central London Community Healthcare NHS Trust
- ◯ Chelsea and Westminster Hospital NHS Foundation Trust
- ◯ Cheshire and Wirral Partnership NHS Foundation Trust
- ◯ Chesterfield Royal Hospital NHS Foundation Trust
- ◯ City Hospitals Sunderland NHS Foundation Trust
- ◯ Cornwall Partnership NHS Foundation Trust
- ◯ Countess Of Chester Hospital NHS Foundation Trust
- ◯ County Durham and Darlington NHS Foundation Trust
- ◯ Coventry and Warwickshire Partnership NHS Trust
- ◯ Croydon Health Services NHS Trust
- ◯ Cumbria Northumberland Tyne and Wear NHS Foundation Trust
- ◯ Dartford and Gravesham NHS Trust
- ◯ Derbyshire Community Health Services NHS Foundation Trust
- ◯ Derbyshire Healthcare NHS Foundation Trust
- ◯ Devon Partnership NHS Trust
- ◯ Doncaster and Bassetlaw Teaching Hospitals NHS Foundation Trust
- ◯ Dorset County Hospital NHS Foundation Trust
- ◯ Dorset Healthcare University NHS Foundation Trust
- ◯ Dudley Integrated Health and Care NHS Trust
- ◯ East and North Hertfordshire NHS Trust
- ◯ East Cheshire NHS Trust
- ◯ East Kent Hospitals University NHS Foundation Trust
- ◯ East Lancashire Hospitals NHS Trust
- ◯ East London NHS Foundation Trust
- ◯ East Midlands Ambulance Service NHS Trust
- ◯ East Of England Ambulance Service NHS Trust
- ◯ East Suffolk and North Essex NHS Foundation Trust
- ◯ East Sussex Healthcare NHS Trust
- ◯ Epsom and St Helier University Hospitals NHS Trust
- ◯ Essex Partnership University NHS Foundation Trust

- ◯ Frimley Health NHS Foundation Trust
- ◯ Gateshead Health NHS Foundation Trust
- ◯ George Eliot Hospital NHS Trust
- ◯ Gloucestershire Care Services NHS Trust
- ◯ Gloucestershire Health and Care NHS Foundation Trust
- ◯ Gloucestershire Hospitals NHS Foundation Trust
- ◯ Great Ormond Street Hospital for Children NHS Foundation Trust
- ◯ Great Western Hospitals NHS Foundation Trust
- ◯ Greater Manchester Mental Health NHS Foundation Trust
- ◯ Guy's and St Thomas' NHS Foundation Trust
- ◯ Hampshire Hospitals NHS Foundation Trust
- ◯ Harrogate and District NHS Foundation Trust
- ◯ Heart Of England NHS Foundation Trust
- ◯ Herefordshire and Worcestershire Health and Care NHS Trust
- ◯ Hertfordshire Community NHS Trust
- ◯ Hertfordshire Partnership University NHS Foundation Trust
- ◯ Homerton University Hospital NHS Foundation Trust
- ◯ Hounslow and Richmond Community Healthcare NHS Trust
- ◯ Hull University Teaching Hospitals NHS Trust
- ◯ Humber Teaching NHS Foundation Trust
- ◯ Imperial College Healthcare NHS Trust
- ◯ Isle of Wight NHS Trust
- ◯ James Paget University Hospitals NHS Foundation Trust
- ◯ Kent and Medway NHS and Social Care Partnership Trust
- ◯ Kent Community Health NHS Foundation Trust
- ◯ Kettering General Hospital NHS Foundation Trust
- ◯ King's College Hospital NHS Foundation Trust
- ◯ Kingston Hospital NHS Foundation Trust
- ◯ Lancashire & South Cumbria NHS Foundation Trust
- ◯ Lancashire Teaching Hospitals NHS Foundation Trust
- ◯ Leeds and York Partnership NHS Foundation Trust
- ◯ Leeds Community Healthcare NHS Trust
- ◯ Leeds Teaching Hospitals NHS Trust
- ◯ Lewisham and Greenwich NHS Trust
- ◯ Lincolnshire Community Health Services NHS Trust
- ◯ Lincolnshire Partnership NHS Foundation Trust
- ◯ Liverpool Heart and Chest NHS Foundation Trust
- ◯ Liverpool University Hospitals NHS Foundation Trust
- ◯ Liverpool Women's NHS Foundation Trust
- ◯ London Ambulance Service NHS Trust
- ◯ London North West University Healthcare NHS Trust
- ◯ Maidstone and Tunbridge Wells NHS Trust
- ◯ Manchester University NHS Foundation Trust
- ◯ Medway NHS Foundation Trust
- ◯ Mersey Care NHS Foundation Trust
- ◯ Mid and South Essex NHS Foundation Trust
- ◯ Mid Cheshire Hospitals NHS Foundation Trust
- ◯ Mid Yorkshire Hospitals NHS Trust
- ◯ Midlands Partnership NHS Foundation Trust
- ◯ Milton Keynes University Hospital NHS Foundation Trust
- ◯ Moorfields Eye Hospital NHS Foundation Trust
- ◯ Norfolk and Norwich University Hospitals NHS Foundation Trust
- ◯ Norfolk and Suffolk NHS Foundation Trust
- ◯ Norfolk Community Health and Care NHS Trust
- ◯ North Bristol NHS Trust
- ◯ North Cumbria Integrated Care NHS Foundation Trust
- ◯ North East Ambulance Service NHS Foundation Trust
- ◯ North East London NHS Foundation Trust
- ◯ North Essex Partnership University NHS Foundation Trust
- ◯ North Middlesex University Hospital NHS Trust

◯ North Staffordshire Combined Healthcare NHS Trust
◯ North Tees and Hartlepool NHS Foundation Trust
◯ North West Ambulance Service NHS Trust
◯ North West Anglia NHS Foundation Trust
◯ North West Boroughs Healthcare NHS Foundation Trust
◯ Northampton General Hospital NHS Trust
◯ Northamptonshire Healthcare NHS Foundation Trust
◯ Northern Care Alliance NHS Foundation Trust
◯ Northern Devon Healthcare NHS Trust
◯ Northern Lincolnshire and Goole NHS Foundation Trust
◯ Northumbria Healthcare NHS Foundation Trust
◯ Nottingham University Hospitals NHS Trust
◯ Nottinghamshire Healthcare NHS Foundation Trust
◯ Oxford Health NHS Foundation Trust
◯ Oxford University Hospitals NHS Foundation Trust
◯ Oxleas NHS Foundation Trust
◯ Pennine Acute Hospitals NHS Trust
◯ Pennine Care NHS Foundation Trust
◯ Poole Hospital NHS Foundation Trust
◯ Portsmouth Hospitals NHS Trust
◯ Project Nightingale NHS Trust
◯ Queen Victoria Hospital NHS Foundation Trust
◯ Robert Jones and Agnes Hunt Orthopaedic and District Hospital NHS Trust
◯ Rotherham Doncaster and South Humber NHS Foundation Trust
◯ Royal Berkshire NHS Foundation Trust
◯ Royal Cornwall Hospitals NHS Trust
◯ Royal Devon and Exeter NHS Foundation Trust
◯ Royal Free London NHS Foundation Trust
◯ Royal Liverpool and Broadgreen University Hospitals NHS Trust
◯ Royal National Orthopaedic Hospital NHS Trust
◯ Royal Papworth Hospital NHS Foundation Trust
◯ Royal Surrey NHS Foundation Trust
◯ Royal United Hospitals Bath NHS Foundation Trust
◯ Salisbury NHS Foundation Trust
◯ Sandwell and West Birmingham Hospitals NHS Trust
◯ Services Hub For East Sussex Healthcare NHS Trust
◯ Sheffield Children's NHS Foundation Trust
◯ Sheffield Health and Social Care NHS Foundation Trust
◯ Sheffield Teaching Hospitals NHS Foundation Trust
◯ Sherwood Forest Hospitals NHS Foundation Trust
◯ Shrewsbury and Telford Hospital NHS Trust
◯ Shropshire Community Health NHS Trust
◯ Solent NHS Trust
◯ Somerset NHS Foundation Trust
◯ South Central Ambulance Service NHS Foundation Trust
◯ South East Coast Ambulance Service NHS Foundation Trust
◯ South London and Maudsley NHS Foundation Trust
◯ South Tees Hospitals NHS Foundation Trust
◯ South Tyneside And Sunderland NHS Foundation Trust
◯ South Tyneside NHS Foundation Trust
◯ South Warwickshire University NHS Foundation Trust
◯ South West London and St George's Mental Health NHS Trust
◯ South West Yorkshire Partnership NHS Foundation Trust
◯ South Western Ambulance Service NHS Foundation Trust
◯ Southern Health NHS Foundation Trust
◯ Southport and Ormskirk Hospital NHS Trust
◯ St George's University Hospitals NHS Foundation Trust
◯ St Helens and Knowsley Teaching Hospitals NHS Trust
◯ Stockport NHS Foundation Trust
◯ Surrey and Borders Partnership NHS Foundation Trust

◯ Surrey and Sussex Healthcare NHS Trust
◯ Sussex Community NHS Foundation Trust
◯ Sussex Partnership NHS Foundation Trust
◯ TAMESIDE AND GLOSSOP INTEGRATED CARE NHS FOUNDATION TRUST
◯ Taunton and Somerset NHS Foundation Trust
◯ Tavistock and Portman NHS Foundation Trust
◯ Esk and Wear Valleys NHS Foundation Trust
◯ The Christie NHS Foundation Trust
◯ The Clatterbridge Cancer Centre NHS Foundation Trust
◯ The Dudley Group NHS Foundation Trust
◯ The Hillingdon Hospitals NHS Foundation Trust
◯ The Newcastle Upon Tyne Hospitals NHS Foundation Trust
◯ The Princess Alexandra Hospital NHS Trust
◯ The Queen Elizabeth Hospital, King's Lynn. NHS Foundation Trust
◯ The Rotherham NHS Foundation Trust
◯ The Royal Bournemouth and Christchurch Hospitals NHS Foundation Trust
◯ The Royal Marsden NHS Foundation Trust
◯ The Royal Orthopaedic Hospital NHS Foundation Trust
◯ The Royal Wolverhampton NHS Trust
◯ The Walton Centre NHS Foundation Trust
◯ Torbay and South Devon NHS Foundation Trust
◯ United Lincolnshire Hospitals NHS Trust
◯ University College London Hospitals NHS Foundation Trust
◯ University Hospital of Derby and Burton NHS Foundation Trust
◯ University Hospital Southampton NHS Foundation Trust
◯ University Hospitals Birmingham NHS Foundation Trust
◯ University Hospitals Bristol and Weston NHS Foundation Trust
◯ University Hospitals Coventry and Warwickshire NHS Trust
◯ University Hospitals Dorset NHS Foundation Trust
◯ University Hospitals Of Leicester NHS Trust
◯ University Hospitals Of Morecambe Bay NHS Foundation Trust
◯ University Hospitals of North Midlands
◯ University Hospitals Plymouth NHS Trust
◯ University Hospitals Sussex NHS Foundation Trust
◯ Walsall Healthcare NHS Trust
◯ Warrington and Halton Hospitals NHS Foundation Trust
◯ West Hertfordshire Teaching Hospitals NHS Trust
◯ West London NHS Trust
◯ West Midlands Ambulance Service University NHS Foundation Trust
◯ West Suffolk NHS Foundation Trust
◯ Whittington Health NHS Trust
◯ WIRRAL COMMUNITY HEALTH AND CARE NHS FOUNDATI[illegible] TRUST
◯ Wirral University Teaching Hospital NHS Foundation Trust
◯ Worcestershire Acute Hospitals NHS Trust
◯ Wigan and Leigh NHS Foundation Trust
◯ Wye Valley NHS Trust
◯ Yeovil District Hospital NHS Foundation Trust
◯ York and Scarborough Teaching Hospitals NHS Foundation Trust
◯ NHS Ayrshire and Arran
◯ NHS Borders
◯ NHS Dumfries and Galloway
◯ NHS Fife
◯ NHS Forth Valley
◯ NHS Grampian

◯ NHS Greater Glasgow and Clyde
◯ NHS Highland
◯ NHS Lanarkshire
◯ NHS Lothian
◯ NHS Orkney
◯ NHS Shetland
◯ NHS Tayside
◯ NHS Western Isles
◯ Aneurin Bevan Health Board
◯ Swansea Bay University Health Board
◯ Cardiff & Vale University Health Board
◯ Hywel Dda Health Board
◯ Cwm Taf Morgannwg Health Board
◯ Betsi Cadwaladr University Health Board
◯ Powys Teaching Health Board
◯ Belfast HSC Trust
◯ South Eastern HSC Trust
◯ Western HSC Trust
◯ Southern HSC Trust
◯ Northern HSC Trust
◯ Other

Other, please specify

____

Name of NHS Hospital Site

This first survey you complete should cover the main site within your trust unless you have a co-lead completing this already. A separate survey should then also be completed for any other sites within the trust that provide surgical services with anaesthetic support that are >1 mile away from the main site. For any questions or further advice please contact us on: pbm.raft@gmail.com

____

Does this site offer day-case surgery ONLY?

◯ Yes
◯ No

How many inpatient beds does this site have?

You can often find this answer on the trust website (answer must be as a number not text)

____

Activity Information

Approximate number of elective operations per year at your hospital

Please answer to the nearest 50-100 cases (answer must be as a number not text)

____

Which surgical specialties does this hospital site provide? (please tick all that apply)	☐ Obstetrics<br>☐ Major Trauma Centre<br>☐ Vascular<br>☐ Cardiac<br>☐ Thoracic<br>☐ Elective Orthopaedics<br>☐ Orthopaedic Trauma<br>☐ Transplant Surgery<br>☐ Major Urology Cancer Resections<br>☐ Major Gynaecology Cancer resections<br>☐ Upper GI<br>☐ Lower GI<br>☐ Hepatobiliary<br>☐ Neurosurgery<br>☐ Major head and neck cancer resections<br>☐ Burns & plastics<br>☐ None of the above
Is there an Emergency Department on site?	◯ Yes<br>◯ No
Is there an Intensive Care Unit on site?	◯ Yes<br>◯ No
Are there dedicated emergency theatres on site?	◯ Yes<br>◯ No
Does this site have a blood bank?	◯ Yes<br>◯ No

Uptake and use of electronic data

Does your NHS Trust have an Electronic Medication Record?	◯ Yes<br>◯ No
If yes, which Electronic Medication Record (EMR) does your trust use?	◯ Allscripts (Oasis/ Sunrise)<br>◯ Atos (Sema-Helix)<br>◯ Cerner (Millennium/ FirstNet)<br>◯ DXC was CSC (Lorenzo/EDIS/i.PM)<br>◯ EMIS (Symphony)<br>◯ Epic<br>◯ Ideagen (Patient First)<br>◯ IMS Maxims<br>◯ Intersystems (TrakCare)<br>◯ Meditech<br>◯ NerveCentre<br>◯ Servelec (Oceano)<br>◯ Silverlink (iCS/PCS)<br>◯ System C (Medway/ Sigma)<br>◯ Other
If Other, please specify	__________________________________
Does your NHS Trust have electronic notes?	◯ Yes<br>◯ No

If yes, which electronic notes system does your site use?	◯ Allscripts (Oasis/ Sunrise)<br>◯ Atos (Sema-Helix)<br>◯ Cerner (Millennium/ FirstNet)<br>◯ was CSC (Lorenzo/EDIS/i.PM)<br>◯ EMIS (Symphony)<br>◯ Epic<br>◯ Ideagen (Patient First)<br>◯ IMS Maxims<br>◯ Intersystems (TrakCare)<br>◯ Meditech<br>◯ NerveCentre<br>◯ Servelec (Oceano)<br>◯ Silverlink (iCS/PCS)<br>◯ System C (Medway/ Sigma)<br>◯ Other
If Other, please specify	__________________________

Intra-operative Documentation

	Electronic	Paper	Both
How is the anaesthetic chart documented?	◯	◯	◯
How are intra-operative medication prescriptions documented?	◯	◯	◯
How are intra-operative blood product prescriptions documented?	◯	◯	◯

Intra-operative Blood Management

Does this site have access to point of care Hb measurements in theatre suites? (examples include blood gas analyser, HemoCue device)	◯ Yes<br>◯ No
If yes, do the point-of-care results link to the electronic system in real time?<br><br>(by this we mean once the point of care test is done does the result link to the electronic health records for the patient)	◯ Yes, all point-of-care tests link to the electronic system<br>◯ No, none of the point-of-care tests link to the electronic system<br>◯ Depends, some link up & some do not<br>◯ N/A, no electronic health records at this site
How are red cells ordered intraoperatively? (tick all that apply)	☐ Phone call<br>☐ Written request<br>☐ Electronic request

Do you you have the facility for electronic remote issue?

(This is a process for obtaining blood units from a blood fridge positioned remotely from the blood bank. It is often done by submitting an electronic query to the blood bank regarding suitability of the patient for electronic issue and then going through a procedure for selecting the correct unit and printing a compatibility label.)

- ◯ Yes
- ◯ No

Anaemia Referral Pathway for Elective Surgery

This section of the survey refers to elective care only (not emergency surgery or trauma services)

At which point of the surgical pathway do patients who fulfil the NICE preoperative testing criteria usually have a full blood count at this site? (tick all that apply)

(Link to summary of guidance of when FBC should be taken from Centre of Perioperative Care Guideline for the Mangement of Anaemia in the Perioperative Pathway: https://cpoc.org.uk/sites/cpoc/files/documents/2022-09/6.%20CPOC_AnaemiaGuideline_Stepsforeachpatient.pdf)

- ☐ At referral for surgery (e.g. from primary care)
- ☐ First surgical consultation
- ☐ When booked or listed for surgery
- ☐ Pre-operative assessment clinic (separate visit to when patient listed for surgery) >6 weeks prior to surgery
- ☐ Pre-operative assessment clinic (separate visit to when patient listed for surgery) < 6 weeks prior to surgery
- ☐ Day of admission for surgery
- ☐ Other
- ☐ N/A site only does minor surgery

If other, please specify

____

Typically what proportion of patients referred for surgery who fulfil the NICE preoperative testing criteria have their full blood count (FBC) taken either at referral to surgery or at first surgical consultation?

(Link to summary of guidance of when FBC should be taken from Centre of Perioperative Care Guideline for the Mangement of Anaemia in the Perioperative Pathway: https://cpoc.org.uk/sites/cpoc/files/documents/2022-09/6.%20CPOC_AnaemiaGuideline_Stepsforeachpatient.pdf)

- ◯ less than 50% of patients
- ◯ between 50-80% of patients
- ◯ >80% of patients
- ◯ N/A site only does minor surgery

Is there a formal elective anaemia referral pathway that patients with anaemia can be referred to prior to surgery?

- ◯ Yes, one formal pathway that covers all surgical specialties
- ◯ Yes, but different surgical specialties have different formal pathways
- ◯ Informal pathways that depend on local knowledge of processes rather than accessible protocol/guidelines
- ◯ No pathway

What is the haemoglobin threshold for referring patients to anaemia pathways?

(Note 120g/L is equivalent to 12g/dL as 130g/L is equivalent to 13g/dL)

- ◯ < 130g/L (all patients)
- ◯ < 120g/L females & < 130g/L males
- ◯ Other

Other, please specify

____

Which specialty manages perioperative anaemia referral pathways? (tick all that apply)

- ☐ Anaesthesia
- ☐ Surgery
- ☐ Haematology
- ☐ Other

Other, please specify

____

Typically what proportion of patients with newly identified anaemia, who are planned to undergo a surgical procedures with anticipated moderate to high blood loss (>500ml or >10% total blood volume), are assessed for the cause of their anaemia prior to surgery at this site?

- ◯ less than 50% of patients
- ◯ between 50-80% of patients
- ◯ >80% of patients
- ◯ This site does not do surgical cases where there is moderate to high anticipated/expected blood loss

When patients are found to be anaemic please select from the list below the tests that are done routinely to investigate the cause of anaemia at this site (either review protocol or review current practice)?
(Note: this list will only appear for sites that do surgical procedures with anticipated moderate to high blood loss)

	Yes	No	Unsure
Serum Ferritin	◯	◯	◯
Transferrin saturation	◯	◯	◯
CRP	◯	◯	◯
Renal function	◯	◯	◯
B12/folate	◯	◯	◯
Reticulocyte count	◯	◯	◯
Reticulocyte haemoglobin content (CHr)	◯	◯	◯

Which professional group(s) decide if anaemia treatment should be initiated at this site? (tick all that apply)

- ☐ Anaesthetists
- ☐ Preoperative specialist nurses
- ☐ Surgeons
- ☐ Haematologists
- ☐ Anaemia specialist nurses
- ☐ Other

If other, please specify

____

If oral iron is prescribed, what is the dosing regimen as per your site's local guidelines? (tick all that apply)

- ☐ Three times a day dosing
- ☐ Twice a day dosing
- ☐ Once a day dosing
- ☐ Alternate day dosing
- ☐ No local hospital guidance on perioperative oral iron dosing
- ☐ Other

If other, please specify

____

If IV iron is prescribed, what is the most common product used?

- ◯ Ferinject (ferric carboxymaltose)
- ◯ Monofer (ferric derisomaltose)
- ◯ Venofer (iron sucrose)
- ◯ CosmoFer (iron dextran)
- ◯ Diafer (ferric derisomaltose)
- ◯ Other
- ◯ Site does not use IV iron

Where are preoperative intravenous iron infusions for elective surgical patients performed? (tick all that apply)

- ☐ Outpatient setting
- ☐ Primary care setting
- ☐ Medical day case unit/ambulatory unit
- ☐ Surgical day case unit/ambulatory unit
- ☐ Perioperative monitored area (e.g. recovery)
- ☐ Labour ward
- ☐ Other

If other, please specify

____

Typically what proportion of patients have a repeat haemoglobin check after iron therapy has been established prior to having an elective procedure?

- ◯ less than 50% of patients
- ◯ between 50-80% of patients
- ◯ >80% of patients

Tranexamic Acid (TXA)

This section refers to the use of tranexamic acid in adult elective & emergency surgical services

---

Is prophylactic TXA use on surgical safety checklists &/or theatre safety briefings for all surgical specialties when moderate to high blood loss (>500ml or >10% total blood volume) is possible/anticipated?

- ◯ Yes
- ◯ No
- ◯ N/A this site does not do operations where expected blood loss is >500ml (e.g. some day case surgery units)

If no, for which of the following specialties is this in place? (tick all that apply)

- ☐ Obstetrics
- ☐ Major Trauma Centre
- ☐ Vascular
- ☐ Cardiac
- ☐ Thoracic
- ☐ Elective Orthopaedics
- ☐ Orthopaedic Trauma
- ☐ Transplant Surgery
- ☐ Major Urology Cancer Resections
- ☐ Major Gynaecology Cancer resections
- ☐ Upper GI
- ☐ Lower GI
- ☐ Hepatobiliary
- ☐ Neurosurgery
- ☐ Major head and neck cancer resections
- ☐ Burns & plastics
- ☐ None of the above

What is the site's policy regarding intravenous TXA for patients undergoing surgical procedures with anticipated moderate to high (>500ml or >10% total blood volume) blood loss?

- ◯ Offer TXA & policy states defined inclusion & exclusion criteria (such as specific contraindications)
- ◯ Offer TXA & policy does not state exclusion criteria (administration at clinician discretion)
- ◯ No local policy guidance for this

In practice do patients consistently/routinely get offered prophylactic intravenous TXA if expected blood loss is >500ml (or >10% total blood volume) at this site (with the exception of obstetrics where TXA is often offered when blood loss reaches 1000ml)?

(Routinely refers to 'care that is provided regularly', contraindications include recent stroke or recent myocardial infarction)

- ◯ Yes across all surgical specialties
- ◯ Specific surgical specialties only
- ◯ Not routinely considered/offered in any surgical specialties

Which surgical specialties routinely use prophylactic TXA if expected blood loss is >500ml (or >10% total blood volume) and there are no contraindications?

(contraindications include recent stroke or recent myocardial infarction)

- ☐ Obstetrics
- ☐ Major Trauma Centre
- ☐ Vascular
- ☐ Cardiac
- ☐ Thoracic
- ☐ Elective Orthopaedics
- ☐ Orthopaedic Trauma
- ☐ Transplant Surgery
- ☐ Major Urology Cancer Resections
- ☐ Major Gynaecology Cancer resections
- ☐ Upper GI
- ☐ Lower GI
- ☐ Hepatobiliary
- ☐ Neurosurgery
- ☐ Major head and neck cancer resections
- ☐ Burns & plastics
- ☐ None of the above

	Electronic	Paper	Both	Not applicable
How is intraoperative INTRAVENOUS TXA (usually given by anaesthetists) documented in the patient notes?	◯	◯	◯	◯
How is intraoperative TOPICAL TXA (usually given by surgeons at surgical site) documented in the patient notes?	◯	◯	◯	◯

What doses of prophylactic INTRAVENOUS TXA are typically given at your site? (tick all that apply)

- ☐ 500mg stat dose
- ☐ 1g stat dose
- ☐ 10-15mg/kg stat dose
- ☐ 15-30mg/kg stat dose
- ☐ 1g stat dose followed by infusion
- ☐ 10-15mg/kg stat dose followed by infusion
- ☐ Not applicable

What dose of intraoperative TOPICAL TXA is typically prescribed when used at your site? (tick all that apply)

- ☐ Up to 1g
- ☐ 1-2g
- ☐ 2-3g
- ☐ Unsure
- ☐ Not applicable

Intraoperative Cell Salvage and resources available

This section refers to adult elective & emergency surgical services

What is your site's policy regarding indications for cell salvage use in non-obstetric cases? (tick all that apply)

- ☐ Use when anticipated/expected intraoperative blood loss is moderate (>500ml)
- ☐ Use when anticipated/expected intraoperative blood loss is high (e.g. >1000ml)
- ☐ Use when cell salvage can be expected to reduce the likelihood of allogeneic (donor) red cell transfusion
- ☐ Use when cell salvage can be expected to reduce the likelihood of severe postoperative anaemia
- ☐ Use when anticipated/expected blood loss is moderate/high and patient refusing allogenic (donor) transfusion
- ☐ Use when anticipated/expected blood loss is moderate/high and patient has complex blood matching requirements (e.g. rare antibodies)
- ☐ No local policy on cell salvage, used at clinician discretion
- ☐ No local policy on cell salvage, not applicable to this site

Is the use of cell salvage avoided at your site in any of the following scenarios?

	Avoided	Not avoided	No site policy	Unsure
Cancer surgery	○	○	○	○
Infected surgical field	○	○	○	○
Sepsis	○	○	○	○
Haemoglobinopathies (e.g. sickle cell disease)	○	○	○	○

What is your site's policy regarding cell salvage use for obstetric cases? (tick all that apply)

- ☐ All elective regardless of bleeding risk
- ☐ All emergency regardless of bleeding risk
- ☐ Elective cases at risk of PPH
- ☐ Emergency cases at risk of PPH
- ☐ If PPH occurs
- ☐ In patients with anaemia
- ☐ In patients who refuse transfusion of blood products
- ☐ In patients with complex transfusion requirements (e.g. unusual antibodies, irradiated or CMV negative blood requirements)
- ☐ No local policy on cell salvage, used at clinician discretion
- ☐ Not applicable to this site

Where is cell salvaged blood administration documented? (tick all that apply)

- ☐ Anaesthetic chart (electronic)
- ☐ Anaesthetic chart (paper)
- ☐ Operation note (electronic)
- ☐ Operation note (paper)
- ☐ Blood prescription record (electronic)
- ☐ Blood prescription record (paper)
- ☐ Medication prescription record (electronic)
- ☐ Medication prescription record (paper)
- ☐ Other

How many cell salvage machines are available at this site?	◯ 0<br>◯ 1<br>◯ 2<br>◯ 3<br>◯ 4<br>◯ 5<br>◯ 6<br>◯ 7<br>◯ 8<br>◯ 9<br>◯ 10<br>◯ >10
How many rapid infusers are available at this site?	◯ 0<br>◯ 1<br>◯ 2<br>◯ 3<br>◯ 4<br>◯ 5<br>◯ 6<br>◯ 7<br>◯ 8<br>◯ 9<br>◯ 10<br>◯ >10
Is point of care coagulation testing (e.g. TEG/ROTEM) available in at least one theatre department/environment?	◯ Yes<br>◯ No
Is point of care coagulation testing (e.g. TEG/ROTEM) available for use during cardiac surgery?<br><br>(by available we mean that there is a point of care machine with the relevant theatre department and that after a blood sample is taken from a patient point of care testing can reasonably expected to be initiated within 5 minutes)	◯ Yes<br>◯ No<br>◯ Not applicable to this site
Is point of care coagulation testing (e.g. TEG/ROTEM) available for use during major obstetric haemorrhage?<br><br>(by available we mean that there is a point of care machine with the relevant theatre department and that after a blood sample is taken from a patient point of care testing can reasonably expected to be initiated within 5 minutes)	◯ Yes<br>◯ No<br>◯ Not applicable to this site
Is there a blood fridge per theatre department at this site?	◯ Yes<br>◯ No
Is data on blood use fed back to clinicians/clinical teams/groups of surgeons/individual surgeons?<br><br>Please clarify this with your departmental transfusion lead or perioperative anaemia lead prior to answering this question if you are unsure	◯ Yes<br>◯ No

If yes, who is this for?

- ☐ Individual anaesthetists
- ☐ Individual surgeon
- ☐ Departmental - anaesthetists
- ☐ Departmental - surgeons

If yes, how frequent is this feedback?

- ◯ Weekly
- ◯ Monthly
- ◯ 2-4 times a year
- ◯ Annually
- ◯ Other
- ◯ Don't know

Please discuss the following answers with your hospital site's transfusion department manager

Often internal and external audits of theatre blood use is managed by the hospital blood bank in liason with the transfusion lead for your department. Either your departmental transfusion lead &/or hospital transfusion department manager should be able to help you answer these questions.

How is data provided for routine INTERNAL audit?

(Internal audit is that which occurs within the hospital site or trust)

- ◯ Largely Paper based data collection
- ◯ Electronic patient records (EPR) data but this is rarely possible/often incomplete
- ◯ Electronic patient records (EPR) data and this is often possible
- ◯ Electronic data supplemented by LIMS data and/or EBTMS (blood transfusion) data
- ◯ No routine internal audit

How is data provided for routine EXTERNAL audit?

(External audit is when a hospital site/trust contributes to national audits, for example the National Comparative Audit of Blood Transfusion)

- ◯ Largely Paper based data collection
- ◯ Electronic patient records (EPR) data but this is rarely possible/often incomplete
- ◯ Electronic patient records (EPR) data and this is often possible
- ◯ Electronic data supplemented by LIMS data and/or EBTMS (blood transfusion) data
- ◯ No routine external audit

Is data on blood usage available to clinicians to look up in real time?

- ◯ Yes
- ◯ No

Does your hospital site use a bedside electronic blood transfusion system?

- ◯ Yes
- ◯ No

Please provide the name of the system

- ◯ BloodTrack Tx
- ◯ Blood 360
- ◯ EPR system
- ◯ Other

Other, please specify

____

What percentage of blood SAMPLES for group & save and cross matching are taken using a bedside electronic system?

0 50 100

(Place a mark on the scale above)

What percentage of transfusions are ADMINISTERED using a bedside electronic system?

0 50 100

(Place a mark on the scale above)

If blood products are ordered by electronic request, are the latest relevant parts of the FBC or coagulation results provided during the ordering process?

(For example: whilst requesting blood products there is a prompt or information box that appears with relevant results prior to you finishing the request designed to prevent unessesary ordering)

◯ Yes
◯ No

Individual anaesthetist survey

Have you distributed the individual anaesthetist survey amongst colleagues at your site?

◯ Yes
◯ No

RAFT feedback and Site Lead names for certificates

We would really like to understand your exeriences on how you found this project and if this is something RAFT should continue to work towards providing on a regular basis.

We would also like to confirm with you who the lead / co-leads are for this site and how names should be displayed on certificates and collaborator lists so that we can correctly recognise your contribution. Any names you put on this form will only be used for certification and collaborator list purposes.

If you would like to be kept up to speed with all things RAFT, including POPPY which will start later this year sign up to our mailing list on our website: https://www.raftrainees.org/contact-us

	Strongly agree	Agree	Neutral	Disagree	Strongly disagree
This project has helped achieve my curriculum and/or career goals	◯	◯	◯	◯	◯
RAFT and TRNs should continue to provide similar short snapshot project opportunities	◯	◯	◯	◯	◯

	Very easy	Easy	Just right	Difficult	Very difficult
As site lead how challenging was this project?	◯	◯	◯	◯	◯

Please tell us in you own words how your experience could have been made better

____

Site lead / co-leads full name(s) to be put on certificates and collaborator lists for any future publications

(please put one name per line)
