# Supplementary material for: Provision of peri‐operative patient blood management strategies in the UK: a national survey of practice
Source: Anaesthesia. 2025 Mar 12;80(7):781–9. doi: 10.1111/anae.16579 (PMC12171794; doi:10.1111/anae.16579)
Supplement: Supplementary file 4 — Table S1. Benchmarking standards with mapped results. Table S2. Checklist for Reporting Of Survey Studies (CROSS). Table S3. Surgical specialties. Table S4. Organisational survey questions and responses for questions relating to peri‐operative anaemia pathways for elective surgery. Table S5. Organisational survey questions relating to tranexamic acid use. Table S6. Organisational questions relating to cell salvage. [file ANAE-80-781-s004.docx]

**Table S1:** Benchmarking standards with mapped results

**Pre-operative**

| **Pre-operative theme** | **Benchmarking standards source** | **Guidance (as described in source)** | **Main results** | **Survey Primary benchmarks and summary opinion of adherence adequacy (by BTRU researchers)** |
| --- | --- | --- | --- | --- |
| Anaemia pathways | CPOC recommendations 2022 | Availability of hospital guideline for detection and management of anaemia applicable to the perioperative setting. | Seventy percent (86/123) of sites reported implementation of a formal elective anaemia pathway as recommended by Centre for Perioperative Care [10]; 54% (66/123) reported a universal pathway to cover all surgical specialities offered locally; and 16% (20/123) reported surgical specialty specific pathways.  Sixty-nine percent (85/123) of hospitals reported local guidance for oral iron supplementation. However, a variety of iron regimens were reported, including most commonly, once daily dosing at 48% (41/85) sites; but other sites reported policies including alternate day dosing (33%, 28/85); twice per day dosing (28%, 24/85); and three times per day dosing (16%, 14/85). Intravenous iron administration was reported as used by 92% (113/123) of sites. | Survey main benchmark: Provision of local pathways for iron optimisation: Inadequate |
|  | GIRFT | Establish oral and IV iron pathways in primary and secondary care with agreed shared responsibilities. |  | Survey main benchmark: Provision of local policies for oral iron: inadequate |
| Assessing for anaemia | GIRFT | Establish early access to haemoglobin levels through primary care and preoperative assessment clinics. | Screening for anaemia was reported to occur at several time points during the patient pre-operative pathway (Table 1 and online Supporting Information Table S1). The most common assessments were reported in a pre-operative assessment clinic > six weeks prior to surgery 71% (87/123) sites followed by in pre-operative assessment clinics < six weeks prior to surgery 67% (83/123). There was overlap with 57 sites who selected both these options. The overall responses suggest that 55% (68/123) of sites did not meet NICE guidance on timing of full blood count [21] (online Supporting Information Table S4), and reveals missed early opportunities for checking haemoglobin, such as point of referral for surgery; first surgical consultation; or when listing for surgery, as set out in recommendations [21, 25] | Survey main benchmark: Timing of anaemia testing in pathways: inadequate |
|  | NICE guidance NG45 | FBC pre-operatively:  Intermediate surgery & ASA 3-4  All major surgery |  |  |
|  | CPOC recommendations 2022 | Hb required for a diagnosis of anaemia |  |  |
|  | CPOC Standards of Care 2022 | All patients referred for surgery who fulfil the NICE preoperative testing criteria should have a full blood count (FBC) at referral to surgery or at first surgical consultation. |  |  |
| Assessing for cause of anaemia | GIRFT | (A low haemoglobin measurement should trigger simultaneous access to haematinics to assess cause of preoperative anaemia.) | Only 15% (19/123) of sites routinely performed all Centre for Perioperative Care suggested blood tests [10] to further identify causes of pre-operative anaemia in patients diagnosed (see Figure 3 to show proportional use of blood tests by site). Whilst the majority of sites regularly checked ferritin (89%, 110/123), transferrin saturation (79%, 97/123) and B12 and Folate (85% 105/123), reticulocyte haemoglobin content was only regularly checked in 20% (24/123) of sites. When sites estimated proportion of anaemic patients undergoing these further investigations; 48% (59/123) of sites indicated less than 80% patients received further investigation with 26% (32/123) of sites reporting it was undertaken in less than 50% of patients. Referral thresholds for anaemia by haemoglobin varied between hospital sites. Over two-thirds of sites (70%, 86/123) described referral pathways with specific thresholds; and in these cases, 48% (41/86) reported a threshold of <130 g.l^-1^ for both females and males, 36% (31/86) a threshold of <130 g.l^-1^ for males and <120 g.l^-1^ for females and 16% (14/86) used other referral thresholds. |  |
|  | CPOC recommendations | Assess for causes of newly identified anaemia if patient undergoing surgical procedures with anticipated moderate-to-high (>500ml) blood loss.26 |  |  |
| Provision of IV iron | NICE Guidance NG24 | Consider intravenous iron before or after surgery for patients who:  have iron-deficiency anaemia and cannot tolerate or absorb oral iron, or are unable to adhere to oral iron treatment | Intravenous iron administration was reported as used by 92% (113/123) of sites. Ferric carboxymaltose was the most frequent formulation used (54%, 61/113 of sites), followed by ferric derisomaltose (41%, 47/113). Most sites reported that iron infusions were typically administered in medical ambulatory units or outpatient settings. |  |
| Anaemia leadership | CPOC 2023 | Appoint a clinical lead for perioperative patients with anaemia (this may be the same lead as for diabetes or frailty in the perioperative setting, see previous CPOC guidance) | Anaesthetists were the most common professional group who manage formal peri-operative anaemia pathways, as reported by 90% (77/86) of sites. |  |

Intraoperative

| **Intra-operative theme** | **Benchmarking standards source** | **guidance** | **results** |  |
| --- | --- | --- | --- | --- |
| **Administration of tranexamic acid** | NICE QS 138 2016 | Adults who are having surgery and are expected to have moderate blood loss are offered tranexamic acid | Table 3 provides a summary of responses regarding availability of local policies for the use of intraoperative tranexamic acid. Eleven sites (9%, 11/123) reported no indication for use of tranexamic acid as they did not perform surgery associated with moderate to high blood loss [9]. Less than half of hospital sites (44%, 49/112) reported a policy for the use of peri-operative intravenous tranexamic acid. Out of the hospitals who reported a tranexamic acid policy, 43% (21/49) reported that their policies indicated an exclusion criterion such as stroke or recent myocardial infarction (online Supporting Information Table S5). Of the 91% (112/123) of sites that performed surgery associated with moderate to high blood loss, only 18% (20/112) included tranexamic acid during theatre safety briefings. Prophylactic tranexamic acid was not routinely offered at 17% (19/112) of sites but despite gaps in formal policy it was routinely used across all surgical specialties at 32% (36/112) of sites and in specific surgical specialties at 51% (57/112) of sites. The most commonly reported dose of tranexamic acid used was 1g intravenously (see online Supporting Information Table S5). Differences in rates of routine tranexamic acid use depending on surgical specialty are shown in Table 3. | Survey main benchmark: Provision of local policies for tranexamic acid: Inadequate |
|  | NICE Guidance NG24 | Offer tranexamic acid to adults undergoing surgery who are expected to have at least moderate blood loss (greater than 500 ml). For advice on using tranexamic acid in primary hip, knee and shoulder replacement |  |  |
|  | CPOC recommendations | Make appropriate use of anti-fibrinolytics (ie Tranexamic acid). See NICE guidelines on this topic  Consider antifibrinolytic (ie tranexamic acid) if expected blood loss >500ml  Adult patients having major in-patient surgery should receive 1 gram of tranexamic acid prior to skin incision to reduce major surgical bleeding and reduce the need for blood transfusion, whether they have anaemia or are at risk  TXA Contraindicated if recent stroke or Myocardial Infarction (as risk of Venous ThromboEmbolism VTE) |  |  |
|  | The Joint Royal Colleges Tranexamic Acid in Surgery Implementation Group | Tranexamic acid should be considered in all adults having in-patient surgery  1 gram of TXA should be given by slow intravenous injection at the start and end of surgery. The first dose should be given just prior to skin incision and the last dose given just after skin closure |  |  |
| **Cell Salvage** | Association of Anaesthetists 2018 | The use of cell salvage is recommended when it can be expected to reduce the likelihood of allogeneic (donor) red cell transfusion and/or severe postoperative anaemia.  We recommend that cell salvage equipment and staff trained to operate it be immediately available 24 h a day in hospitals undertaking surgery where blood loss is a recognised complication. | Local policies for the use of cell salvage were not present at 59% (73/123) of sites overall, and not present at 34% (26/76) of sites offering obstetric services. At sites with local polices the most common organisational level indication for cell salvage was patient refusal of allogeneic transfusion for both obstetric and non-obstetric patients (see online Supporting Information Table S6). | Survey main benchmark: Provision of local policies for cell salvage: inadequate |
|  | GIRFT 2021 | Ensure cell salvage systems are available when required in all surgical specialities through infrastructure, staff training and audit of use. |  |  |
| **Point of Care Hb Testing** | BTRU expert consensus | Sites undergoing surgery with potential for major blood loss should have availability of:point of care hb and coagulation testing | Point of care coagulation testing was available at 50% (62/123) of sites. |  |
| **Rapid infuser devices** | BTRU expert consensus | Sites undergoing surgery with potential for major blood loss should have availability of: rapid infusion device | There was variation in the availability of rapid infusers dependent on specialty and hospital size (Table 4) |  |
| **Ability to remote issue blood** | BTRU expert consensus | Sites undergoing surgery with potential for major blood loss should have ability to remote issue blood | Thirty seven percent (45/123) of sites reported availability of remote blood issuance |  |

**Table S2** Checklist for Reporting Of Survey Studies (CROSS)

| **Section/topic** | **Item** | **Item description** | **Reported on page #** | **Comments** |
| --- | --- | --- | --- | --- |
| **Title and abstract** | | |  |  |
| Title and abstract | 1a | State the word “survey” along with a commonly used term in title or abstract to introduce the study’s design. | 1 |  |
|  | 1b | Provide an informative summary in the abstract, covering background, objectives, methods, findings/results, interpretation/discussion, and conclusions. | 2 |  |
| **Introduction** | | |  |  |
| Background | 2 | Provide a background about the rationale of study, what has been previously done, and why this survey is needed. | 3 |  |
| Purpose/aim | 3 | Identify specific purposes, aims, goals, or objectives of the study. | 3 |  |
| **Methods** | | |  |  |
| Study design | 4 | Specify the study design in the methods section with a commonly used term (e.g., cross-sectional or longitudinal). | 4 |  |
|  | 5a | Describe the questionnaire (e.g., number of sections, number of questions, number and names of instruments used). | 4 |  |
| Data collection methods | 5b | Describe all questionnaire instruments that were used in the survey to measure particular concepts. Report target population, reported validity and reliability information, scoring/classification procedure, and reference links (if any). | 4 |  |
|  | 5c | Provide information on pretesting of the questionnaire, if performed (in the article or in an online supplement). Report the method of pretesting, number of times questionnaire was pre-tested, number and demographics of participants used for pretesting, and the level of similarity of demographics between pre-testing participants and sample population. | 4 |  |
|  | 5d | Questionnaire if possible, should be fully provided (in the article, or as appendices or as an online supplement). | 4  Supporting info |  |
| Sample characteristics | 6a | Describe the study population (i.e., background, locations, eligibility criteria for participant inclusion in survey, exclusion criteria). | 4-5  Fig 1, fig 2 | Our survey encompass practice across the full range of NHS services where major inpatient surgery is undertaken, with spread of geographical location, hospital size and specialty provision described in results |
|  | 6b | Describe the sampling techniques used (e.g., single stage or multistage sampling, simple random sampling, stratified sampling, cluster sampling, convenience sampling). Specify the locations of sample participants whenever clustered sampling was applied. | n/a | RAFT networks were used in order to avoid the pitfalls of convenient sampling and hence avoid reporting bias, and in the region without RAFT representation, College Tutors and Heads of Schools of Anaesthesia were contacted |
|  | 6c | Provide information on sample size, along with details of sample size calculation. | 4-5 | All UK NHS hospital sites were included. Invitations to take part were shared amongst RAFT to regional Trainee Research Networks (TRNs), RAFT mailing list and by contacting College Tutors and Heads of the Schools of Anaesthesia in regions without RAFT representation |
|  | 6d | Describe how representative the sample is of the study population (or target population if possible), particularly for population-based surveys. | 5  Figure 1, 2  Table 4 | Our survey encompass practice across the full range of NHS services where major inpatient surgery is undertaken, with spread of geographical location, hospital size and specialty provision described in results |
| Survey  administration | 7a | Provide information on modes of questionnaire administration, including the type and number of contacts, the location where the survey was conducted (e.g., outpatient room or by use of online tools, such as SurveyMonkey). | 4 |  |
|  | 7b | Provide information of survey’s time frame, such as periods of recruitment, exposure, and follow-up days. | 4-5 |  |
|  | 7c | Provide information on the entry process:  –>For non-web-based surveys, provide approaches to minimize human error in data entry.  –>For web-based surveys, provide approaches to prevent “multiple participation” of participants. | 4- 5 |  |
| Study preparation | 8 | Describe any preparation process before conducting the survey (e.g., interviewers’ training process, advertising the survey). | 4-5 |  |
| Ethical considerations | 9a | Provide information on ethical approval for the survey if obtained, including informed consent, institutional review board [IRB] approval, Helsinki declaration, and good clinical practice [GCP] declaration (as appropriate). | 4 |  |
|  | 9b | Provide information about survey anonymity and confidentiality and describe what mechanisms were used to protect unauthorized access. | 4 |  |
| Statistical  analysis | 10a | Describe statistical methods and analytical approach. Report the statistical software that was used for data analysis. | 5 |  |
|  | 10b | Report any modification of variables used in the analysis, along with reference (if available). | n/a |  |
|  | 10c | Report details about how missing data was handled. Include rate of missing items, missing data mechanism (i.e., missing completely at random [MCAR], missing at random [MAR] or missing not at random [MNAR]) and methods used to deal with missing data (e.g., multiple imputation). | 5, 9 | Missing or incomplete data for organisational survey responses was checked with sites. Incomplete surveys after this process, were excluded to minimise missing data. |
|  | 10d | State how non-response error was addressed. | 5, 9  Fig 1 | Missing or incomplete data for organisational survey responses was checked with sites. Incomplete surveys after this process, were excluded to minimise missing data.  Limitations include those common to all surveys, that they may not reflect actual practice or the actual practice of non-respondents |
|  | 10e | For longitudinal surveys, state how loss to follow-up was addressed. | n/a |  |
|  | 10f | Indicate whether any methods such as weighting of items or propensity scores have been used to adjust for non-representativeness of the sample. | n/a |  |
|  | 10g | Describe any sensitivity analysis conducted. | n/a |  |
| **Results** | | |  |  |
| Respondent characteristics | 11a | Report numbers of individuals at each stage of the study. Consider using a flow diagram, if possible. | 5 fig 1 |  |
|  | 11b | Provide reasons for non-participation at each stage, if possible. | 5 fig 1 |  |
|  | 11c | Report response rate, present the definition of response rate or the formula used to calculate response rate. | 5 |  |
|  | 11d | Provide information to define how unique visitors are determined. Report number of unique visitors along with relevant proportions (e.g., view proportion, participation proportion, completion proportion). | 4-5 | there was only one username/password per site and therefore only one survey per site could be recorded by the site lead (no duplication). To address the unique visitor aspect. |
| Descriptive  results | 12 | Provide characteristics of study participants, as well as information on potential confounders and assessed outcomes. | 5 table 2 |  |
| Main findings | 13a | Give unadjusted estimates and, if applicable, confounder-adjusted estimates along with 95% confidence intervals and p-values. | 5-7 |  |
|  | 13b | For multivariable analysis, provide information on the model building process, model fit statistics, and model assumptions (as appropriate). | n/a |  |
|  | 13c | Provide details about any sensitivity analysis performed. If there are considerable amount of missing data, report sensitivity analyses comparing the results of complete cases with that of the imputed dataset (if possible). | n/a |  |
| **Discussion** | | |  |  |
| Limitations | 14 | Discuss the limitations of the study, considering sources of potential biases and imprecisions, such as non-representativeness of sample, study design, important uncontrolled confounders. | 9-10 |  |
| Interpretations | 15 | Give a cautious overall interpretation of results, based on potential biases and imprecisions and suggest areas for future research. | 8-10 |  |
| Generalizability | 16 | Discuss the external validity of the results. | 8-9 |  |
| **Other sections** | | |  |  |
| Role of funding source | 17 | State whether any funding organization has had any roles in the survey’s design, implementation, and analysis. | 10 |  |
| Conflict of interest | 18 | Declare any potential conflict of interest. | 10 |  |
| Acknowledgements | 19 | Provide names of organizations/persons that are acknowledged along with their contribution to the research. | 10  Supporting information S1 |  |

**Table S3** Surgical Specialties

| **Surgical specialty provision at responding sites** | |
| --- | --- |
| Lower GI | 101 |
| Elective orthopaedics | 91 |
| Orthopaedic trauma | 80 |
| Obstetrics | 76 |
| Upper GI | 74 |
| Major gynaecology cancer resections | 63 |
| Major urology cancer resections | 56 |
| Major head and neck resections | 38 |
| HPB | 36 |
| Vascular | 33 |
| Burns & plastics | 23 |
| Cardiac | 22 |
| Thoracic | 22 |
| Transplant surgery | 19 |
| Major trauma centre | 18 |
| Neurosurgery | 15 |

**Table S4:** Organisational survey questions and responses for questions relating to peri-operative anaemia pathways for elective surgery

| **At which point of the surgical pathway do patients who fulfil the NICE preoperative testing criteria usually have a full blood count at this site? (tick all that When booked or listed for surgery apply)** | | | | | | | | | |
| --- | --- | --- | --- | --- | --- | --- | --- | --- | --- |
|  | | | | | | Number of sites | | | |
| At referral for surgery | | | | | | 16 | | | |
| First surgical consultation | | | | | | 13 | | | |
| When booked or listed for surgery | | | | | | 17 | | | |
| Pre-operative assessment clinic (separate visit to when patient listed for surgery) >6 weeks prior to surgery | | | | | | 87 | | | |
| Pre-operative assessment clinic (separate visit to when patient listed for surgery) < 6 weeks prior to surgery | | | | | | 83 | | | |
| Day of admission for surgery | | | | | | 13 | | | |
| Other | | | | | | 5 | | | |
| n/a minor surgery only | | | | | | 5 | | | |
| **When patients are found to be anaemic please select from the list below the tests that are done routinely to investigate the cause of anaemia at this site (either review protocol or review current practice)?** | | | | | | | | | |
|  | Serum Ferritin | Transferrin saturation | CRP | Renal function | Vitamin B12 & folate | | | Reticulocyte count | Reticulocyte haemoglobin content (CHr) |
| Yes | 110 | 97 | 74 | 89 | 105 | | | 40 | 24 |
| No | 3 | 14 | 30 | 15 | 8 | | | 58 | 69 |
| Unsure | 1 | 3 | 10 | 10 | 1 | | | 16 | 21 |
| Missing | 9 | 9 | 9 | 9 | 9 | | | 9 | 9 |
| **Which professional group(s) decide if anaemia treatment should be initiated at this site? (tick all that apply)** | | | | | | | | | |
|  | | | | | | Number of sites | | | |
| Anaesthetists | | | | | | 108 | | | |
| Preoperative specialist nurses | | | | | | 70 | | | |
| Surgeons | | | | | | 40 | | | |
| Haematologists | | | | | | 33 | | | |
| Anaemia specialist nurses | | | | | | 21 | | | |
| Other | | | | | | 10 | | | |
| **If oral iron is prescribed, what is the dosing regimen as per your site's local guidelines? (tick all that apply)** | | | | | | | | | |
|  | | | | | | Number of sites | | | |
| Three times a day dosing | | | | | | 15 | | | |
| Twice a day dosing | | | | | | 24 | | | |
| Once a day dosing | | | | | | 41 | | | |
| Alternate day dosing | | | | | | 28 | | | |
| No local hospital guidance on perioperative oral iron dosing | | | | | | 39 | | | |
| Other | | | | | | 12 | | | |
| **If IV iron is prescribed, what is the most common product used?** | | | | | | | | | |
|  | | | | | | Number of sites | | | |
| Ferinject (ferric carboxymaltose) | | | | | | 61 | | | |
| Monofer (ferric derisomaltose) | | | | | | 47 | | | |
| Venofer (iron sucrose) | | | | | | 2 | | | |
| CosmoFer (iron dextran) | | | | | | 3 | | | |
| Diafer (ferric derisomaltose) | | | | | | 0 | | | |
| Other | | | | | | 0 | | | |
| Site does not use IV iron | | | | | | 10 | | | |
| **Where are preoperative intravenous iron infusions for elective surgical patients performed? (tick all that apply)** | | | | | | | | | |
|  | | | | | | | Number of sites | | |
| Outpatient setting | | | | | | | 45 | | |
| Primary care setting | | | | | | | 7 | | |
| Medical day case unit/ambulatory unit | | | | | | | 56 | | |
| Surgical day case unit/ambulatory unit | | | | | | | 35 | | |
| Perioperative monitored area (e.g. recovery) | | | | | | | 10 | | |
| Labour ward | | | | | | | 10 | | |
| Other | | | | | | | 12 | | |

**Table S5:** Organisational survey questions relating to Tranexamic acid use

| **Is prophylactic TXA use on surgical safety checklists &/or theatre safety briefings for all surgical specialties when moderate to high blood loss (>500ml or >10% total blood volume) is possible/anticipated?** | |
| --- | --- |
|  | Number of sites |
| Yes | 20 |
| No | 92 |
| N/A this site does not do operations where expected blood loss is >500ml (e.g. some day case surgery units) | 11 |
| **What is the site's policy regarding intravenous TXA for patients undergoing surgical procedures with anticipated moderate to high (>500ml or >10% total blood volume) blood loss?** | |
|  | Number of sites |
| Offer TXA & policy states defined inclusion & exclusion criteria (such as specific contraindications) | 21 |
| Offer TXA & policy does not state exclusion criteria (administration at clinician discretion) | 28 |
| No local policy guidance for this | 63 |
| N/A this site does not do operations where expected blood loss is >500ml (e.g. some day case surgery units) | 11 |
| **In practice do patients consistently/routinely get offered prophylactic intravenous TXA if expected blood loss is >500ml (or >10% total blood volume) at this site (with the exception of obstetrics where TXA is often offered when blood loss reaches 1000ml)?** | |
|  | Number of sites |
| Yes, across all surgical specialties | 36 |
| Specific surgical specialties only | 57 |
| Not routinely considered/offered in any surgical specialties | 19 |
| N/A this site does not do operations where expected blood loss is >500ml (e.g. some day case surgery units) | 11 |
| **What doses of prophylactic INTRAVENOUS TXA are typically given at your site? (tick all that apply)** | |
|  | Number of sites |
| 500mg stat dose | 2 |
| 1g stat dose | 105 |
| 10-15mg/kg stat dose | 9 |
| 15-30mg/kg stat dose | 3 |
| 1g stat dose followed by infusion | 19 |
| 10-15mg/kg stat dose followed by infusion | 6 |
| Not applicable | 8 |
| **What dose of intraoperative TOPICAL TXA is typically prescribed when used at your site? (tick all that apply)** | |
|  | Number of sites |
| Up to 1g | 7 |
| 1-2g | 7 |
| 2-3g | 4 |
| Unsure | 53 |
| Not applicable | 53 |

**Table S6:** Organisational questions relating to cell salvage

| **What is your site's policy regarding indications for cell salvage use in non-obstetric cases? (tick all that apply)** | **N** |
| --- | --- |
| Use when anticipated/expected intraoperative blood loss is moderate (>500ml) | 23 |
| Use when anticipated/expected intraoperative blood loss is high (e.g. >1000ml) | 36 |
| Use when cell salvage can be expected to reduce the likelihood of allogeneic (donor) red cell transfusion | 33 |
| Use when cell salvage can be expected to reduce the likelihood of severe postoperative anaemia | 22 |
| Use when anticipated/expected blood loss is moderate/high and patient refusing allogenic (donor) transfusion | 41 |
| Use when anticipated/expected blood loss is moderate/high and patient has complex blood matching requirements (e.g. rare antibodies) | 34 |
| No local policy on cell salvage, used at clinician discretion | 30 |
| No local policy on cell salvage, not applicable to this site | 43 |
| **What is your site's policy regarding cell salvage use for obstetric cases? (tick all that apply)** | **N** |
| All elective regardless of bleeding risk | 2 |
| All emergency regardless of bleeding risk | 4 |
| Elective cases at risk of PPH | 39 |
| Emergency cases at risk of PPH | 29 |
| If PPH occurs | 21 |
| In patients with anaemia | 17 |
| In patients who refuse transfusion of blood products | 49 |
| In patients with complex transfusion requirements (e.g. unusual antibodies, irradiated or CMV negative blood requirements) | 31 |
| No local policy on cell salvage, used at clinician discretion | 26 |
| Not applicable to this site | 52 |
